# Crystal structure of 4-(di­methyl­amino)­pyridinium *cis*-di­aqua­bis­(oxalato-κ^2^ *O*,*O*′)ferrate(III) hemihydrate

**DOI:** 10.1107/S2056989015013213

**Published:** 2015-07-15

**Authors:** Edith Dimitri Djomo, Frédéric Capet, Justin Nenwa, Michel M. Bélombé, Michel Foulon

**Affiliations:** a Department of Inorganic Chemistry, University of Yaounde 1, POB 812 Yaounde, Cameroon; b Unité de Catalyse et de Chimie du Solide, UMR 8181, École Nationale Supérieure de Chimie de Lille, Université Lille-1, 59650 Villeneuve d’Ascq Cedex, France; c UFR de Physique, Université Lille-1, 59650 Villeneuve d’Ascq Cedex, France

**Keywords:** crystal structure, 4-(di­methyl­amino)­pyridine, bis­(oxalate)ferrate(III) complex, hybrid salt, hydrogen bonding

## Abstract

The Fe^III^ ions in the hybrid title salt, (C_7_H_11_N_2_)[Fe(C_2_O_4_)_2_(H_2_O)_2_]·0.5H_2_O, show a distorted octa­hedral coordination environment, with four O atoms from two chelating oxalate dianions and two O atoms from two *cis* aqua ligands. The average Fe—O(oxalate) bond length [2.00 (2) Å] is shorter than the average Fe—O(water) bond length [2.027 (19) Å]. The ionic components are connected *via* inter­molecular N—H⋯O and O—H⋯O hydrogen bonds into a three-dimensional network.

## Abstract 2

In the title compound, the Fe^III^ atom is bonded to four O atoms from two chelating oxalate dianions and two O atoms from two *cis* aqua ligands. It has a slightly distorted octa­hedral coordination geometry. In the crystal, N—H⋯O and O—H⋯O hydrogen bonds play an important role in the structural self-assembly.

## Chemical context   

Over the past years, the design and synthesis of organic–inorganic hybrid salts have attracted much attention not only because of their fascinating network topologies, but also to obtain a better understanding of the correlations between their structural and physical properties (Bloomquist *et al.*, 1981 ▸; Geiser *et al.*, 1987 ▸; Pardo *et al.*, 2012 ▸). In this context, the bis-oxalato complexes of transition metals, [*M* ^III^(C_2_O_4_)_2_(H_2_O)_2_]^−^, are extremely versatile building blocks for the synthesis of organic–inorganic hybrid salts. Although several salts of general formula *A*[*M* ^III^(C_2_O_4_)_2_(H_2_O)_2_]·*x*H_2_O (*A* ^+^ = aromatic iminium cation, 0≤*x*≤1) have been explored to date (Bélombé *et al.*, 2009 ▸; Nenwa *et al.*, 2010 ▸; Chérif *et al.*, 2011 ▸; Chérif, Abdelhak *et al.*, 2012 ▸; Chérif, Zid *et al.*, 2012 ▸; Nenwa *et al.*, 2012**a* ▸,b* ▸; Dridi *et al.*, 2013 ▸; Bebga *et al.*, 2013 ▸), the predictable and consistent formation of networks is still in its infancy. In most cases, the network topologies are influenced by the organic counter-cations, metal coordination spheres, pH values, guest mol­ecules and the crystallization solvent. So far, most of the self-assembly processes involving anionic species, [*M* ^III^(C_2_O_4_)_2_(H_2_O)_2_]^−^, and aromatic iminium cations have led to salts with *trans*-di­aqua­bis­(oxalate)metallate(III) complex anions (Bélombé *et al.*, 2009 ▸, Nenwa *et al.*, 2010 ▸, 2012*a* ▸; Chérif, Zid *et al.*, 2012 ▸; Dridi *et al.*, 2013 ▸; Gouet *et al.*, 2013 ▸). The *cis* configuration of the complex anion [*M* ^III^(C_2_O_4_)_2_(H_2_O)_2_]^−^ is less common in the literature, and has been observed in salts with 2-amino-5-chloro­pyridinium (Chérif, Abdelhak *et al.*, 2012 ▸) or with pyridinium (Nenwa *et al.*, 2012*b* ▸) as aromatic iminium cations. In this work, we extend this family of salts involving the complex anion [*M* ^III^(C_2_O_4_)_2_(H_2_O)_2_]^−^ in its *cis*-configuration by reporting the structural characterization of the title compound with composition (C_7_H_11_N_2_)[Fe(C_2_O_4_)_2_(H_2_O)_2_]·0.5H_2_O.
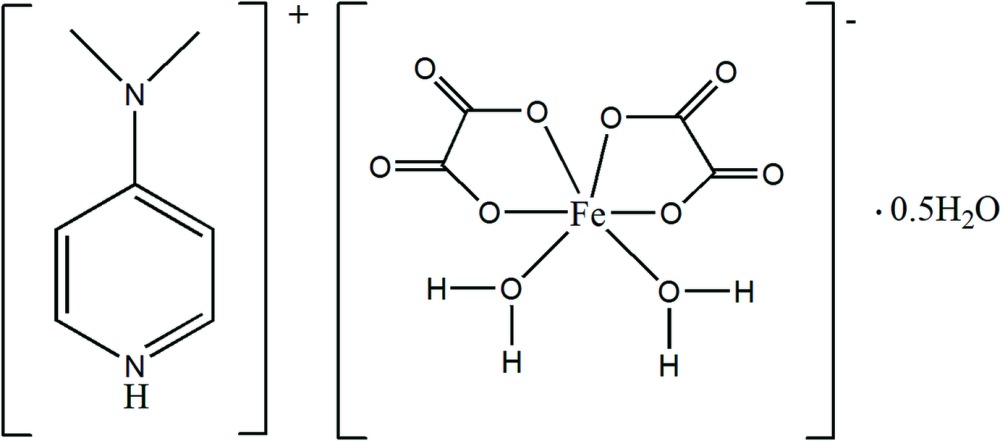


## Structural commentary   

**Figure 1:**
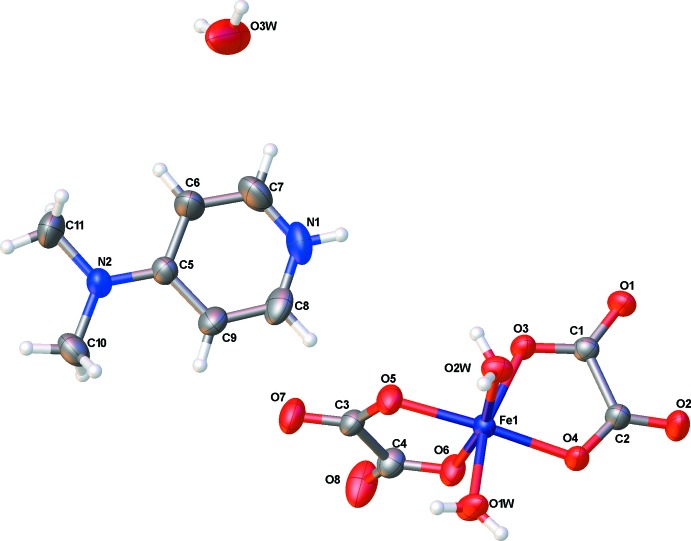
View of the mol­ecular components of (I), showing the atom-numbering scheme. Displacement ellipsoids are drawn at the 50% probability level.

The asymmetric unit of the title compound shown in Fig. 1 ▸ consists of one protonated 4-(di­methyl­amino)­pyridine mol­ecule (C_7_H_11_N_2_)^+^, one anionic complex [Fe(C_2_O_4_)_2_(H_2_O)_2_]^−^ in a *cis*-aqua configuration and one-half solvent water mol­ecule. Atom O3*W* of this water mol­ecule of solvation lies on a crystallographic twofold rotation axis. The main geometric parameters of the (C_7_H_11_N_2_)^+^ cation are in agreement with those found in a similar salt with the same cationic entity (Nenwa *et al.*, 2010 ▸). The iron(III) site in the complex anion has a distorted octa­hedral coordination environment built up by two O atoms (O1*W*, O2*W*) from two *cis-*aqua ligands and four O atoms (O3, O4, O5, O6) from two chelating oxalate dianions. The average Fe—O_(oxalate)_ bond length [2.00 (2) Å] is shorter than the average Fe—O_(water)_ bond length [2.027 (19) Å]. The bond lengths in the [Fe(C_2_O_4_)_2_(H_2_O)_2_]^−^ anion are similar to those observed in homologous compounds with a *cis*-aqua configuration of the [*M* ^III^(C_2_O_4_)_2_(H_2_O)_2_]^−^ anionic units (Chérif, Abdelhak *et al.*, 2012 ▸; Nenwa *et al.*, 2012*b* ▸).

## Supra­molecular features   

**Figure 2:**
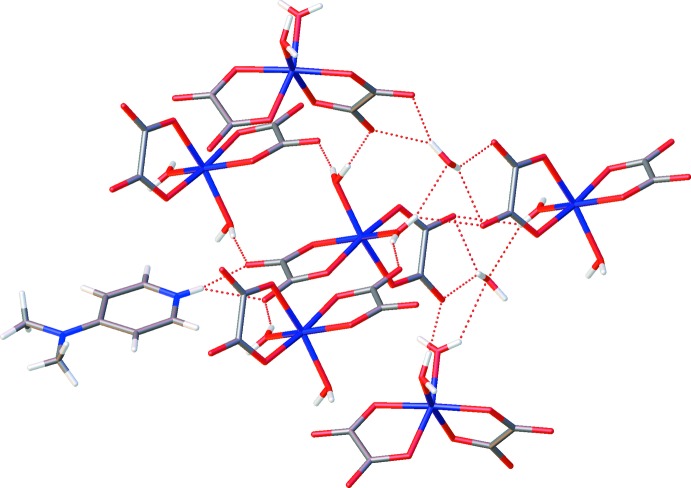
The environment of the [Fe(C_2_O_4_)_2_(H_2_O)_2_]^−^ octa­hedron. Dashed lines denote hydrogen bonds.

**Figure 3:**
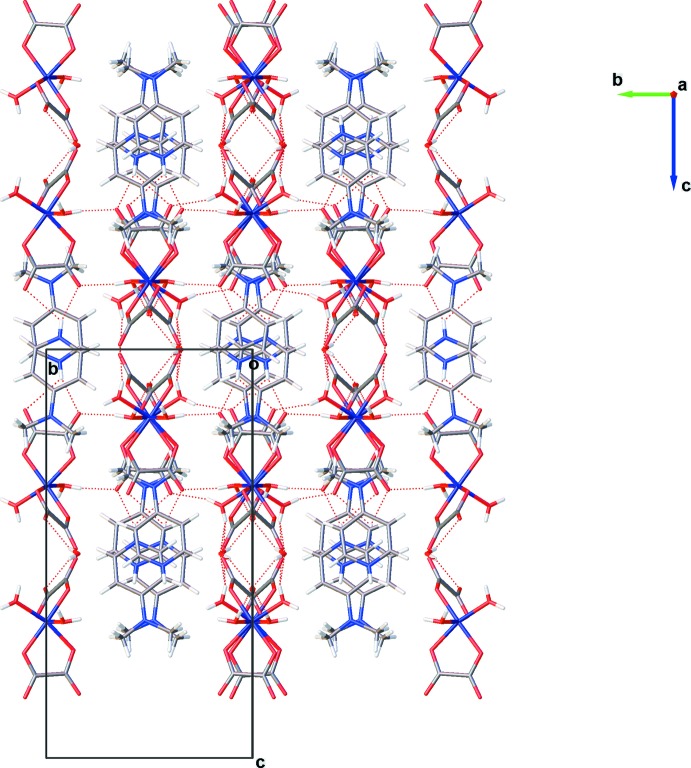
A (100) projection of the crystal structure of the title compound. Hydrogen bonds are shown as dashed lines.

Within the crystal packing, the charged components are connected by an extensive hydrogen-bonding network. Hydrogen bonds of the type O—H⋯O involving coordinating water mol­ecules as donor groups and auxilliary O atoms of the oxalate dianions as acceptor groups inter­connect neighboring [Fe(C_2_O_4_)_2_(H_2_O)_2_]^−^ anionic units (Table 1 ▸, Fig. 2 ▸). Together with the relatively weaker N—H⋯O hydrogen bonds of the protonated imine N atoms of the 4-(di­methyl­amino)­pyridine mol­ecules to the oxalate dianions, a three-dimensional framework is formed (Table 1 ▸, Fig. 3 ▸).

## Synthesis and crystallization   

The salt Fe(NO_3_)_3_·6H_2_O (1 mmol, 400 mg) was dissolved in 20 ml of water, leading to a yellowish solution. This solution was added in successive small portions in 30 ml of a mixture of oxalic acid (2 mmol, 253 mg) and 4-(di­methyl­amino)­pyridine (1 mmol, 122 mg) with stirring at 323 K for 2 h. The resulting greenish solution was left at room temperature; crystals suitable for X-ray diffraction were obtained after two weeks upon slow evaporation.

## Refinement   

Crystal data, data collection and structure refinement details are summarized in Table 2 ▸. H atoms bonded to C and N atoms were placed at geometrically calculated positions and refined using a riding model. C—H distances were fixed at 0.93 and 0.96 Å for aromatic and methyl C atoms, respectively. The N—H distance was fixed at 0.86 Å. The *U*_iso_(H) values were equal to 1.2 and 1.5 times *U*_eq_ of the corresponding C(*sp* ^2^) and C(*sp* ^3^) atoms, and 1.2 times *U*_eq_ of the N atom. All water H atoms were located from a difference-Fourier map and refined with soft restraints on the O—H and H⋯H distances [O—H = 0.82 (2) and H⋯H = 1.30 (4) Å] with *U*_iso_(H) = 1.5*U*_eq_(O).

## Supplementary Material

Crystal structure: contains datablock(s) I. DOI: 10.1107/S2056989015013213/vn2095sup1.cif

Structure factors: contains datablock(s) I. DOI: 10.1107/S2056989015013213/vn2095Isup2.hkl

CCDC reference: 1400489

Additional supporting information:  crystallographic information; 3D view; checkCIF report

## Figures and Tables

**Table 1:** Hydrogen-bond geometry (, )

*D*H*A*	*D*H	H*A*	*D* *A*	*D*H*A*
O1*W*H1*WA*O2^i^	0.81(2)	1.94(2)	2.720(3)	162(4)
O1*W*H1*WB*O7^ii^	0.77(2)	2.40(3)	2.988(3)	134(4)
O1*W*H1*WB*O3*W* ^iii^	0.77(2)	2.34(3)	2.9699(19)	139(4)
O2*W*H2*WA*O8^iv^	0.82(2)	1.84(2)	2.664(3)	176(3)
O2*W*H2*WB*O1^v^	0.83(2)	1.88(2)	2.702(2)	171(3)
N1H1O1^v^	0.86	2.11	2.931(3)	160
N1H1O2^v^	0.86	2.46	3.043(3)	125
O3*W*H3*W*O7^vi^	0.84(2)	2.36(5)	3.040(2)	138(6)
O3*W*H3*W*O8^vi^	0.84(2)	2.09(5)	2.782(3)	140(6)

Symmetry codes: (i) 
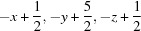
; (ii) 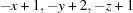; (iii) 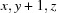; (iv) 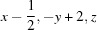; (v) 
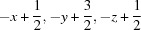
; (vi) 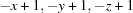.

**Table 2:** Experimental details

Crystal data	
Chemical formula	(C_7_H_11_N_2_)[Fe(C_2_O_4_)_2_(H_2_O)_2_]0.5H_2_O
*M*_r_	800.22
Crystal system, space group	Monoclinic, *I*2/*a*
Temperature (K)	296
*a*, *b*, *c* ()	14.7960(7), 10.4422(4), 21.7751(10)
()	108.352(3)
*V* (^3^)	3193.2(2)
*Z*	4
Radiation type	Mo *K*
(mm^1^)	1.00
Crystal size (mm)	0.26 0.22 0.13

Data collection	
Diffractometer	Bruker APEXII CCD
Absorption correction	Multi-scan (*SADABS*; Bruker, 2014 ▸)
*T*_min_, *T*_max_	0.679, 0.746
No. of measured, independent and observed [*I* > 2(*I*)] reflections	48070, 4880, 3435
*R*_int_	0.044
(sin /)_max_ (^1^)	0.715

Refinement	
*R*[*F* ^2^ > 2(*F* ^2^)], *wR*(*F* ^2^), *S*	0.042, 0.123, 1.06
No. of reflections	4880
No. of parameters	239
No. of restraints	8
H-atom treatment	H atoms treated by a mixture of independent and constrained refinement
_max_, _min_ (e ^3^)	0.85, 0.58

Computer programs: *APEX2* and *SAINT* (Bruker, 2014 ▸), *SHELXS97* (Sheldrick, 2008 ▸), *SHELXL2014* (Sheldrick, 2015 ▸), *OLEX2* (Dolomanov *et al.*, 2009 ▸), *publCIF* (Westrip, 2010 ▸) and *PLATON* (Spek, 2009 ▸).

## supplementary crystallographic information

### Crystal data

**Table:**

(C_7_H_11_N_2_)[Fe(C_2_O_4_)_2_(H_2_O)_2_]·0.5H_2_O	*F*(000) = 1648
*M**_r_* = 800.22	*D*_x_ = 1.665 Mg m^−^^3^
Monoclinic, *I*2/*a*	Mo *K*α radiation, λ = 0.71073 Å
*a* = 14.7960 (7) Å	Cell parameters from 9923 reflections
*b* = 10.4422 (4) Å	θ = 2.2–27.3°
*c* = 21.7751 (10) Å	µ = 1.00 mm^−^^1^
β = 108.352 (3)°	*T* = 296 K
*V* = 3193.2 (2) Å^3^	Irregular, yellow
*Z* = 4	0.26 × 0.22 × 0.13 mm

### Data collection

**Table:**

Bruker APEXII CCD diffractometer	3435 reflections with *I* > 2σ(*I*)
Radiation source: sealed X-ray tube	*R*_int_ = 0.044
φ and ω scans	θ_max_ = 30.6°, θ_min_ = 2.8°
Absorption correction: multi-scan (*SADABS2014*; Bruker, 2014)	*h* = −21→21
*T*_min_ = 0.679, *T*_max_ = 0.746	*k* = −14→14
48070 measured reflections	*l* = −31→31
4880 independent reflections	

### Refinement

**Table:**

Refinement on *F*^2^	Primary atom site location: structure-invariant direct methods
Least-squares matrix: full	Secondary atom site location: difference Fourier map
*R*[*F*^2^ > 2σ(*F*^2^)] = 0.042	Hydrogen site location: mixed
*wR*(*F*^2^) = 0.123	H atoms treated by a mixture of independent and constrained refinement
*S* = 1.06	*w* = 1/[σ^2^(*F*_o_^2^) + (0.0528*P*)^2^ + 5.0524*P*] where *P* = (*F*_o_^2^ + 2*F*_c_^2^)/3
4880 reflections	(Δ/σ)_max_ = 0.001
239 parameters	Δρ_max_ = 0.85 e Å^−^^3^
8 restraints	Δρ_min_ = −0.58 e Å^−^^3^

### Special details

**Table:**

Experimental. Absorption correction: SADABS-2014/3 (Bruker, 2014) was used for absorption correction. wR2(int) was 0.0678 before and 0.0491 after correction. The Ratio of minimum to maximum transmission is 0.9094. The λ/2 correction factor is 0.00150.
Geometry. All e.s.d.'s (except the e.s.d. in the dihedral angle between two l.s. planes) are estimated using the full covariance matrix. The cell e.s.d.'s are taken into account individually in the estimation of e.s.d.'s in distances, angles and torsion angles; correlations between e.s.d.'s in cell parameters are only used when they are defined by crystal symmetry. An approximate (isotropic) treatment of cell e.s.d.'s is used for estimating e.s.d.'s involving l.s. planes.

### Fractional atomic coordinates and isotropic or equivalent isotropic displacement parameters (Å^2^)

**Table:**

	*x*	*y*	*z*	*U*_iso_*/*U*_eq_	
Fe1	0.33846 (2)	0.99760 (3)	0.33390 (2)	0.02994 (10)	
O1	0.27198 (13)	0.84143 (16)	0.15711 (7)	0.0393 (4)	
O1W	0.31148 (18)	1.14895 (18)	0.38270 (9)	0.0511 (5)	
H1WA	0.287 (3)	1.216 (2)	0.3669 (16)	0.077*	
H1WB	0.321 (3)	1.132 (4)	0.4186 (10)	0.077*	
O2	0.23808 (16)	1.10293 (17)	0.14845 (8)	0.0524 (5)	
O2W	0.21255 (12)	0.91604 (16)	0.33264 (9)	0.0381 (4)	
H2WA	0.187 (2)	0.941 (3)	0.3590 (13)	0.057*	
H2WB	0.223 (2)	0.8379 (17)	0.3394 (15)	0.057*	
O3	0.33473 (12)	0.86776 (14)	0.26407 (7)	0.0343 (3)	
O4	0.29136 (13)	1.10922 (15)	0.25610 (7)	0.0389 (4)	
O5	0.39926 (12)	0.89364 (16)	0.41206 (8)	0.0398 (4)	
O6	0.47565 (13)	1.04452 (18)	0.34780 (8)	0.0408 (4)	
O7	0.53967 (14)	0.85689 (19)	0.48747 (9)	0.0504 (5)	
O8	0.62163 (14)	1.0053 (2)	0.41370 (12)	0.0625 (6)	
C1	0.29387 (15)	0.9073 (2)	0.20675 (10)	0.0293 (4)	
C2	0.27184 (17)	1.0531 (2)	0.20161 (10)	0.0338 (5)	
C3	0.49036 (17)	0.9055 (2)	0.43727 (11)	0.0335 (5)	
C4	0.53485 (18)	0.9927 (2)	0.39683 (12)	0.0377 (5)	
N1	0.34280 (18)	0.5649 (3)	0.47049 (11)	0.0564 (6)	
H1	0.3107	0.5735	0.4302	0.068*	
N2	0.49507 (17)	0.5221 (2)	0.66339 (10)	0.0442 (5)	
C5	0.44607 (18)	0.5376 (2)	0.60033 (11)	0.0371 (5)	
C6	0.4118 (3)	0.4314 (3)	0.55903 (14)	0.0609 (9)	
H6	0.4241	0.3488	0.5756	0.073*	
C7	0.3617 (3)	0.4484 (4)	0.49606 (15)	0.0683 (10)	
H7	0.3400	0.3771	0.4700	0.082*	
C8	0.3735 (2)	0.6686 (3)	0.50711 (14)	0.0518 (7)	
H8	0.3599	0.7493	0.4885	0.062*	
C9	0.42434 (19)	0.6587 (3)	0.57107 (13)	0.0438 (6)	
H9	0.4448	0.7324	0.5955	0.053*	
C10	0.5313 (2)	0.6307 (3)	0.70562 (14)	0.0521 (7)	
H10A	0.5788	0.6742	0.6919	0.078*	
H10B	0.5591	0.6011	0.7493	0.078*	
H10C	0.4801	0.6885	0.7036	0.078*	
C11	0.5175 (3)	0.3955 (3)	0.69141 (16)	0.0704 (10)	
H11A	0.4600	0.3467	0.6833	0.106*	
H11B	0.5480	0.4030	0.7372	0.106*	
H11C	0.5596	0.3530	0.6723	0.106*	
O3W	0.2500	0.1564 (4)	0.5000	0.117 (2)	
H3W	0.302 (3)	0.115 (5)	0.512 (3)	0.176*	

### Atomic displacement parameters (Å^2^)

**Table:**

	*U*^11^	*U*^22^	*U*^33^	*U*^12^	*U*^13^	*U*^23^
Fe1	0.03909 (18)	0.02585 (16)	0.02228 (15)	−0.00039 (12)	0.00595 (11)	−0.00107 (11)
O1	0.0583 (11)	0.0306 (8)	0.0283 (8)	−0.0001 (7)	0.0126 (7)	−0.0071 (6)
O1W	0.0945 (16)	0.0285 (9)	0.0335 (9)	0.0046 (9)	0.0247 (10)	−0.0040 (7)
O2	0.0879 (15)	0.0359 (9)	0.0250 (8)	0.0134 (9)	0.0059 (9)	0.0014 (7)
O2W	0.0401 (9)	0.0323 (8)	0.0426 (9)	0.0015 (7)	0.0141 (7)	−0.0069 (7)
O3	0.0490 (10)	0.0253 (7)	0.0270 (7)	0.0055 (6)	0.0099 (7)	−0.0007 (6)
O4	0.0615 (11)	0.0244 (8)	0.0252 (7)	0.0070 (7)	0.0058 (7)	−0.0026 (6)
O5	0.0411 (9)	0.0398 (9)	0.0349 (8)	−0.0073 (7)	0.0070 (7)	0.0109 (7)
O6	0.0430 (9)	0.0422 (9)	0.0349 (9)	−0.0037 (7)	0.0089 (7)	0.0125 (7)
O7	0.0505 (11)	0.0505 (11)	0.0431 (10)	0.0023 (8)	0.0047 (8)	0.0186 (8)
O8	0.0355 (10)	0.0863 (17)	0.0660 (14)	0.0012 (10)	0.0163 (9)	0.0341 (12)
C1	0.0349 (11)	0.0266 (10)	0.0281 (10)	0.0003 (8)	0.0121 (8)	−0.0021 (8)
C2	0.0442 (12)	0.0283 (10)	0.0273 (10)	0.0042 (9)	0.0088 (9)	−0.0016 (8)
C3	0.0412 (12)	0.0280 (10)	0.0308 (10)	0.0008 (9)	0.0106 (9)	0.0051 (8)
C4	0.0377 (12)	0.0379 (12)	0.0390 (12)	0.0001 (9)	0.0140 (10)	0.0058 (10)
N1	0.0565 (14)	0.0824 (19)	0.0260 (10)	0.0018 (13)	0.0071 (10)	0.0052 (11)
N2	0.0548 (13)	0.0377 (11)	0.0308 (10)	0.0045 (9)	−0.0001 (9)	−0.0004 (8)
C5	0.0457 (13)	0.0341 (11)	0.0293 (11)	0.0038 (10)	0.0086 (9)	−0.0016 (9)
C6	0.090 (2)	0.0377 (15)	0.0408 (14)	0.0106 (14)	0.0006 (14)	−0.0075 (12)
C7	0.088 (3)	0.063 (2)	0.0414 (16)	0.0035 (18)	0.0019 (15)	−0.0201 (15)
C8	0.0521 (16)	0.0561 (17)	0.0468 (15)	−0.0012 (13)	0.0152 (12)	0.0233 (13)
C9	0.0515 (15)	0.0350 (13)	0.0418 (13)	−0.0050 (10)	0.0101 (11)	0.0070 (10)
C10	0.0519 (16)	0.0573 (17)	0.0395 (13)	−0.0063 (13)	0.0032 (11)	−0.0116 (12)
C11	0.087 (2)	0.0517 (18)	0.0514 (17)	0.0136 (17)	−0.0089 (16)	0.0126 (14)
O3W	0.076 (3)	0.057 (2)	0.154 (4)	0.000	−0.056 (3)	0.000

### Geometric parameters (Å, º)

**Table:**

Fe1—O1W	2.0133 (18)	N1—C7	1.330 (5)
Fe1—O2W	2.0407 (18)	N1—C8	1.337 (4)
Fe1—O3	2.0250 (15)	N2—C5	1.345 (3)
Fe1—O4	1.9927 (16)	N2—C10	1.452 (3)
Fe1—O5	1.9799 (16)	N2—C11	1.450 (4)
Fe1—O6	2.0163 (18)	C5—C6	1.417 (4)
O1—C1	1.235 (3)	C5—C9	1.407 (3)
O1W—H1WA	0.809 (17)	C6—H6	0.9300
O1W—H1WB	0.771 (17)	C6—C7	1.349 (4)
O2—C2	1.223 (3)	C7—H7	0.9300
O2W—H2WA	0.824 (17)	C8—H8	0.9300
O2W—H2WB	0.834 (17)	C8—C9	1.363 (4)
O3—C1	1.272 (3)	C9—H9	0.9300
O4—C2	1.272 (3)	C10—H10A	0.9600
O5—C3	1.291 (3)	C10—H10B	0.9600
O6—C4	1.269 (3)	C10—H10C	0.9600
O7—C3	1.218 (3)	C11—H11A	0.9600
O8—C4	1.226 (3)	C11—H11B	0.9600
C1—C2	1.554 (3)	C11—H11C	0.9600
C3—C4	1.550 (3)	O3W—H3W	0.842 (19)
N1—H1	0.8600		
			
O1W—Fe1—O2W	90.16 (8)	O8—C4—O6	125.9 (2)
O1W—Fe1—O3	163.51 (8)	O8—C4—C3	119.0 (2)
O1W—Fe1—O6	95.03 (9)	C7—N1—H1	119.9
O3—Fe1—O2W	84.35 (7)	C7—N1—C8	120.2 (2)
O4—Fe1—O1W	85.14 (7)	C8—N1—H1	119.9
O4—Fe1—O2W	99.16 (8)	C5—N2—C10	121.7 (2)
O4—Fe1—O3	80.42 (6)	C5—N2—C11	121.2 (2)
O4—Fe1—O6	92.95 (7)	C11—N2—C10	117.1 (2)
O5—Fe1—O1W	95.09 (8)	N2—C5—C6	121.6 (2)
O5—Fe1—O2W	87.05 (7)	N2—C5—C9	122.9 (2)
O5—Fe1—O3	100.13 (7)	C9—C5—C6	115.5 (2)
O5—Fe1—O4	173.78 (7)	C5—C6—H6	119.5
O5—Fe1—O6	80.84 (7)	C7—C6—C5	121.0 (3)
O6—Fe1—O2W	167.19 (7)	C7—C6—H6	119.5
O6—Fe1—O3	93.64 (7)	N1—C7—C6	121.4 (3)
Fe1—O1W—H1WA	126 (3)	N1—C7—H7	119.3
Fe1—O1W—H1WB	110 (3)	C6—C7—H7	119.3
H1WA—O1W—H1WB	123 (3)	N1—C8—H8	119.2
Fe1—O2W—H2WA	118 (2)	N1—C8—C9	121.5 (3)
Fe1—O2W—H2WB	107 (2)	C9—C8—H8	119.2
H2WA—O2W—H2WB	107 (3)	C5—C9—H9	119.8
C1—O3—Fe1	114.30 (13)	C8—C9—C5	120.3 (3)
C2—O4—Fe1	116.08 (14)	C8—C9—H9	119.8
C3—O5—Fe1	116.33 (14)	N2—C10—H10A	109.5
C4—O6—Fe1	114.72 (15)	N2—C10—H10B	109.5
O1—C1—O3	126.2 (2)	N2—C10—H10C	109.5
O1—C1—C2	119.43 (19)	H10A—C10—H10B	109.5
O3—C1—C2	114.39 (17)	H10A—C10—H10C	109.5
O2—C2—O4	126.3 (2)	H10B—C10—H10C	109.5
O2—C2—C1	119.91 (19)	N2—C11—H11A	109.5
O4—C2—C1	113.77 (18)	N2—C11—H11B	109.5
O5—C3—C4	112.84 (19)	N2—C11—H11C	109.5
O7—C3—O5	126.3 (2)	H11A—C11—H11B	109.5
O7—C3—C4	120.9 (2)	H11A—C11—H11C	109.5
O6—C4—C3	115.1 (2)	H11B—C11—H11C	109.5
			
Fe1—O3—C1—O1	−170.06 (19)	O7—C3—C4—O6	−175.6 (2)
Fe1—O3—C1—C2	10.4 (2)	O7—C3—C4—O8	5.0 (4)
Fe1—O4—C2—O2	176.4 (2)	N1—C8—C9—C5	0.1 (4)
Fe1—O4—C2—C1	−3.0 (3)	N2—C5—C6—C7	179.1 (3)
Fe1—O5—C3—O7	174.7 (2)	N2—C5—C9—C8	−179.2 (3)
Fe1—O5—C3—C4	−4.7 (3)	C5—C6—C7—N1	0.1 (6)
Fe1—O6—C4—O8	178.3 (2)	C6—C5—C9—C8	0.0 (4)
Fe1—O6—C4—C3	−1.1 (3)	C7—N1—C8—C9	−0.2 (5)
O1—C1—C2—O2	−4.1 (4)	C8—N1—C7—C6	0.1 (5)
O1—C1—C2—O4	175.3 (2)	C9—C5—C6—C7	−0.2 (5)
O3—C1—C2—O2	175.4 (2)	C10—N2—C5—C6	179.2 (3)
O3—C1—C2—O4	−5.2 (3)	C10—N2—C5—C9	−1.6 (4)
O5—C3—C4—O6	3.8 (3)	C11—N2—C5—C6	1.9 (5)
O5—C3—C4—O8	−175.6 (2)	C11—N2—C5—C9	−178.8 (3)

### Hydrogen-bond geometry (Å, º)

**Table:**

*D*—H···*A*	*D*—H	H···*A*	*D*···*A*	*D*—H···*A*
O1*W*—H1*WA*···O2^i^	0.81 (2)	1.94 (2)	2.720 (3)	162 (4)
O1*W*—H1*WB*···O7^ii^	0.77 (2)	2.40 (3)	2.988 (3)	134 (4)
O1*W*—H1*WB*···O3*W*^iii^	0.77 (2)	2.34 (3)	2.9699 (19)	139 (4)
O2*W*—H2*WA*···O8^iv^	0.82 (2)	1.84 (2)	2.664 (3)	176 (3)
O2*W*—H2*WB*···O1^v^	0.83 (2)	1.88 (2)	2.702 (2)	171 (3)
N1—H1···O1^v^	0.86	2.11	2.931 (3)	160
N1—H1···O2^v^	0.86	2.46	3.043 (3)	125
O3*W*—H3*W*···O7^vi^	0.84 (2)	2.36 (5)	3.040 (2)	138 (6)
O3*W*—H3*W*···O8^vi^	0.84 (2)	2.09 (5)	2.782 (3)	140 (6)

Symmetry codes: (i) −*x*+1/2, −*y*+5/2, −*z*+1/2; (ii) −*x*+1, −*y*+2, −*z*+1; (iii) *x*, *y*+1, *z*; (iv) *x*−1/2, −*y*+2, *z*; (v) −*x*+1/2, −*y*+3/2, −*z*+1/2; (vi) −*x*+1, −*y*+1, −*z*+1.
